# Overexpression of Lysosome-Associated Membrane Protein 1 in Oral Squamous Cell Carcinoma and its Correlation with Tumor Differentiation and Metastasis

**DOI:** 10.22038/IJORL.2021.51683.2772

**Published:** 2022-01

**Authors:** Mohammad Ali Ranjbar, Maryam Jamshidi

**Affiliations:** 1 *Department of Oral and Maxillofacial Pathology, School of Dentistry, Shiraz University of Medical Sciences, Shiraz, Iran.*; 2 *Undergraduate Student, School of Dentistry, Shiraz University of Medical Sciences, Shiraz, Iran.*

**Keywords:** Immunohistochemistry, LAMP1, Oral squamous cell carcinoma

## Abstract

**Introduction:**

The overexpression of lysosome-associated membrane protein 1 (LAMP1) has been demonstrated in different types of cancers, such as ovarian carcinoma, esophageal squamous cell carcinoma, breast cancer, and colorectal carcinoma. Nonetheless, the expression of LAMP1 in oral squamous cell carcinoma (OSCC) has not been investigated yet.

**Materials and Methods:**

This cross-sectional study was conducted on 65 patients with OSCC selected from the Department of Pathology of Shiraz University of Medical Sciences. The control group comprised 55 tissues of normal oral epithelium. The expression of LAMP1 in OSCC tissue samples was assessed using immunohistochemistry (IHC) analysis. The association between LAMP1 and clinicopathological features of patients with OSCC was also evaluated.

**Results:**

The expression of LAMP1 was significantly higher in OSCC tissues, as compared to that in normal tissues (P<0.001). The chi-square analysis indicated that the high LAMP1 expression was correlated with the degree of tumor differentiation and metastasis (P=0.014).

**Conclusions:**

The obtained results pointed to the overexpression of LAMP1 in OSCC, as well as its correlation with tumor grade and metastasis; therefore, LAMP1 might have a role to play in OSCC pathogenesis and could be regarded as an independent prognostic marker in oral squamous cell carcinoma.

## Introduction

Oral cancers are considered one of the leading causes of death with an increasing incidence across the globe (1). The majority of oral cancers histologically originate from squamous cells of the epithelium and are categorized as oral squamous cell carcinoma (OSCC) (2). According to the World Health Organization reports, OSCC is the sixth and tenth most common cancer in men and women, respectively (3). Evaluations are made for early diagnosis and prevention of this deadly cancer to improve the achieved outcome. The conventional treatment methods for OSCC include surgery and radiation therapy. Treatment protocols of the advanced OSCC consist of surgical resection with postoperative radiotherapy (4-6). Despite the advances in diagnostic techniques and treatment, OSCC is a disorder with a high mortality rate, 30% regional or local recurrence, 25% distant metastasis, and a 5-year survival rate of 45-50% (2). Nowadays, diagnosis and treatment of OSCC are based on clinical and histopathological features (7). Recent studies in the field of molecular pathology have introduced more than 1,000 molecular biomarkers, which can demonstrate biological differences between cancers and predict patient outcomes (7,8). In this regard, molecular techniques, such as immunohistochemistry, have been suggested as a valuable tool for the quick diagnosis and management of OSCC (7). 

Lysosome-associated membrane protein-1 (LAMP1) which belongs to the LAMP protein family was initially suggested as a protein of mature dendritic cells (CD208, DC-LAMP) (9). The LAMP1, which is also known as CD107a, is recognized to protect the lysosomal membrane integrity from intracellular proteolysis and maintain the lysosomal acidification (10,11). It has been proposed that the terminal glycan residue, sLeX, on LAMP-1 is involved in cellular adhesion, tumor invasion, and metastasis by binding to E-selectin-expressing endothelial cells (12,13). Although LAMP1 is primarily expressed in the endosome-lysosomal membrane of cells, it could be expressed in the plasma membrane (14,15). Several studies have pointed out that LAMP1 is overexpressed on the cell surface of highly metastatic tumor cells, suggesting the possible role of LAMP1 in cell-cell adhesion and migration of tumor cells(15-17). The molecular analysis of LAMP1 expression has demonstrated that LAMP1 might play a biological role in the progression and development of different cancers, such as colorectal tumors, pancreatic carcinoma, ovarian cancer, breast cancer, and esophageal squamous cell carcinoma (ESCC) (18-22). 

 A previous study investigating the expression of LAMP1 in breast cancers exhibited a higher expression of this protein than corresponding non-cancerous tissues. The association between LAMP1 expression and clinicopathological features of breast cancer, such as histological grade and lymph node metastasis, highlighted that LAMP1 might be a prognostic marker in patients with breast cancer (18). Another investigation into the prognostic value of LAMP1 in esophageal squamous cell carcinoma illustrated the correlation of LAMP1 expression with tumor histological differentiation and patients’ prognosis (21).

To the best of our knowledge, there is no research examining the association of LAMP1 expression with OSCC and clinicopathological factors. In light of the aforementioned issues, the present study aimed to assess the expression of this marker in OSCC, as well as its possible role as a diagnostic and prognostic marker. 

## Materials and Methods

This cross-sectional study was conducted on 65 OSCC tissues from the archive of the Oral Pathology Department of Shiraz Dental School and a control group of 55 cases of normal oral epithelium adjacent to the lesions. Two pathologists reviewed all the samples. This study was reviewed and approved by the Medical Ethics Committee of Shiraz University of Medical Science (IR.SUMS.REC.1398.379). Informed consent was obtained from all patients. Firstly, the Hematoxylin and Eosin (H&E) slides of blocks were investigated, and the cases with a definite diagnosis and adequate cellular tissue were determined for Immunohistochemistry (IHC). The IHC staining was performed using the EnVision Labeled Peroxides System (DAKO, Carpentaria, CA, USA). The prepared sections with 4μ thickness were mounted on slides after deparaffinization and rehydration in graded alcohol. Non-specific binding sites were blocked with 3% hydrogen peroxide in methanol for 30 min. The sections were then incubated with the anti-LAMP1 antibody (Abcam, ab25245) at 4°C overnight. The normal tissues were stained with the same amount of the antibody used to stain tumoral tissues. The primary antibody was eliminated in the negative controls, while human lung tissue sections were prepared as a positive control for LAMP1. Brown membranous and cytoplasmic staining for LAMP1 was considered positive. 

The slides were evaluated under a light microscope (Olympus CX31; Tokyo, Japan) at 400X magnification. The percent of LAMP1 positivity was evaluated as follows: 0, 0-19%; 1, 20-39%; 2, 40-59%, and 3, 60-100%. The intensity of LAMP1 staining was also evaluated as follows: 0= negative, 1=weakly positive, 2=moderately positive, and 3=strongly positive. The sum of the percentage and intensity scores was generated as the final LAMP1 staining score and determined as follows: <3 demonstrating low or no expression and ≥3 displaying high expression (18,23).The data were analyzed in SPSS software (version 20). Mann-Whitney U test was used to compare the expression of LAMP1 between OSCC tissues and normal tissues. The statistically significant differences between LAMP1 expression and clinicopathological parameters were assessed by the Chi-square test. A p-value less than 0.05 was considered statistically significant. 

## Results

**Fig 1:**
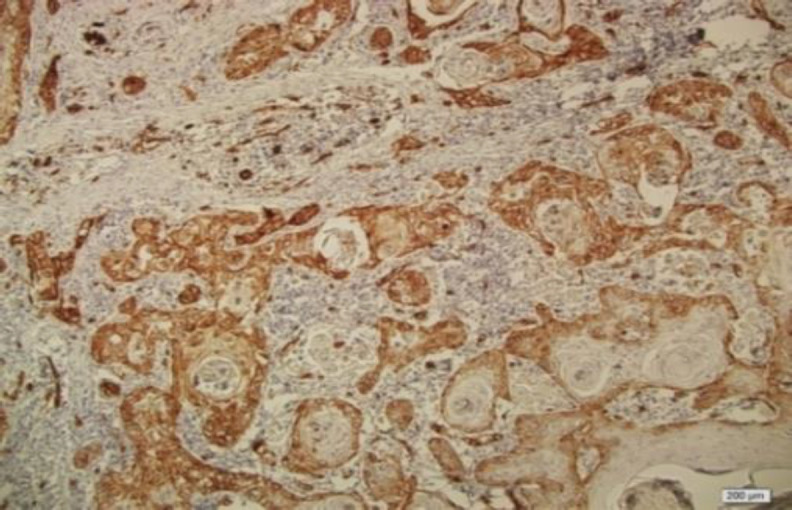
Cytoplasmic and membranous expression of LAMP1 in oral squamous cell carcinoma (200X)

**Fig 2:**
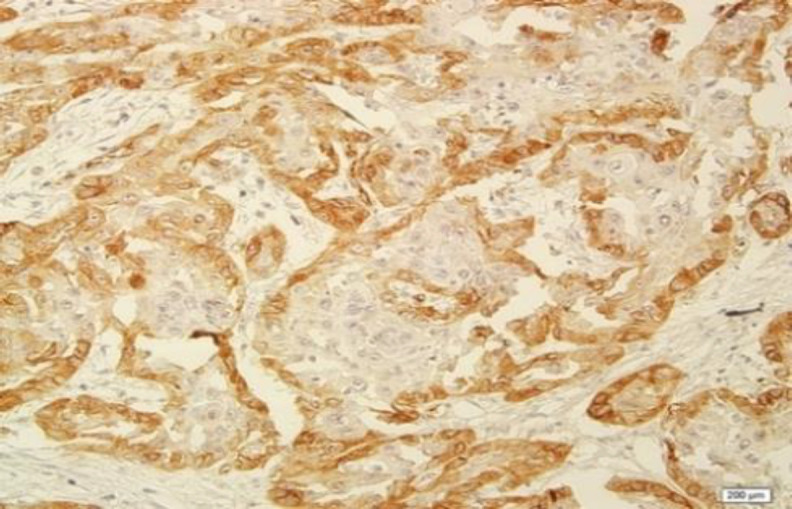
Cytoplasmic and membranous expression of LAMP1 in oral squamous cell carcinoma (400X)

The patients consisted of 22 (33.9 %) females and 43 (66.1%) males with a mean age of 68.5±3.75 years. The control group consisted of 28 (50.9%) females and 27 (49.1%) males with a mean age of 45.6±1.75 years. As illustrated by figures 1 and 2, LAMP1 expression was detected in the cytoplasm and membrane of the epithelial cells of OSCC. The LAMP1 positive expression was detected in 56.9% (37/65) of OSCC samples, compared to 14.5% (8/55) of non-cancerous tissue samples (Table 1). 

**Table 1:** Immunohistochemical staining of LAMP1 protein in OSCC and normal tissue samples

**Samples (n)**	**LAMP1 expression** **Low or no expression N (%) High expression N (%)**	**P-value**
Normal tissue (55)	47 (85.5%) 8 (14.5%)	<0.001
OSCC (65)	28 (43.1%) 37 (56.9%)	

Statistical analysis indicated that the LAMP1 expression level was significantly higher in OSCC samples, as compared to that in normal tissues (P<0.001). 

**Fig 3:**
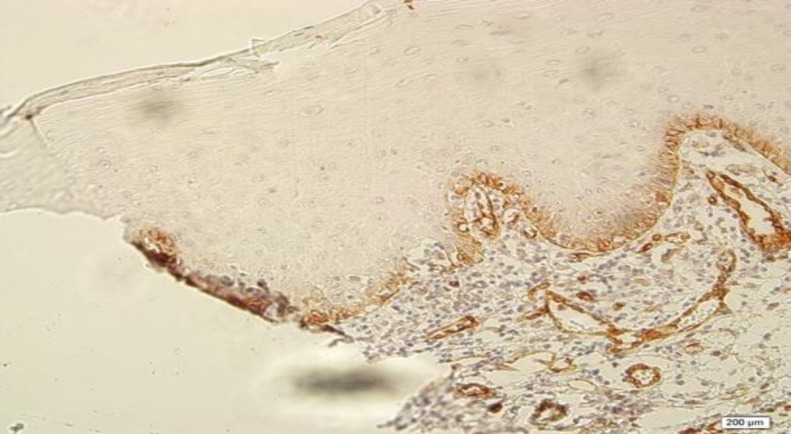
Low expression of LAMP1 in normal oral epithelium (200X)

Normal oral mucosa illustrated LAMP1 expression in the basal layer of epithelium (Figure 3). 

The relationship between the LAMP1 expression and the clinicopathological features of patients was also evaluated.

As displayed in Table 2, increased LAMP1 expression is significantly correlated with histological grade (P= 0.014) and tumor metastasis (P=0.014). Nonetheless, no significant relationship was also found between the LAMP1 expression and other clinical features, such as gender, age, tumor size, as well as the tumor, nodes, and metastases (TNM) stages (P>0.05).

**Table 2:** Association of LAMP1 expression with clinicopathologic characteristics in OSCC

**Variable**	**N (%)**	**Low or no expression** **N (%)**	**High expression** **N (%)**	**P. Value**
Age				
>60 ≤60	40 (61.5) 25 (38.5)	19 (29.2) 9 (13.9)	21 (32.3) 16 (24.6)	0.362
Gender				
M F	43 (66.2) 22 (33.8)	18 (27.7) 10 (15.4)	25 (38.5) 12 (18.4)	0.782
Grade				
Well Moderate poor	22 (33.9) 27 (41.5) 16 (24.6)	15 (23.1) 8 (12.3) 5 (7.7)	7 (10.8) 19 (29.2) 11 (16.9)	0.014
Metastasis				
No Yes	48 (73.9) 17 (26.1)	25 (38.5) 3 (4.6)	23 (35.4) 14 (21.5)	0.014
TNM				
I+II III+Ⅳ	46 (70.8) 19 (29.2)	21 (32.3) 7 (10.8)	25 (38.5) 12 (18.4)	0.514
Tumor size				
T1+T2 T3+T4	37 (56.9) 28 (43.1)	17 (26.1) 11 (17)	20 (30.8) 17 (26.1)	0.591

## Discussion

The OSCC is associated with genetic changes due to the absence of cellular growth control and differentiation mechanism (2). Molecular markers can play a crucial role in the prediction of tumor aggressiveness (4,5). 

The identification of these biological markers, such as LAMP1, increases the ability of the clinical staging system to estimate the prognosis and progression of OSCC. The LAMP1 is a highly glycosylated type 1 transmembrane protein with a large luminal domain (11). An increased expression of LAMP1 has been demonstrated on cells involved in invasive functions, such as natural killer cells, activated cytotoxic T lymphocytes, macrophages, and platelets (15-17).

In the current study, the overexpression of LAMP1 was observed in OSCC, compared to the normal tissues (P<0.001), signifying the role of LAMP1 in the carcinogenesis of OSCC. The results of the present research were in agreement with those of the previous studies illustrating the overexpression of LAMP1 in ovarian cancers and breast tumors, compared to non-cancerous samples (18,22). 

Moreover, the current study revealed that the expression of LAMP1 was significantly associated with histopathological grading and metastasis of tumors. The lower degree of histological differentiation results in higher levels of LAMP1 expression. In accordance with our results, the findings of some studies have pointed to the association between LAMP1 expression and the clinicopathological features of tumors, including histological grade and metastasis (18,21-23). 

A previous study on the immunohistochemical expression of LAMP1 in ESCC suggested that the LAMP1expression was significantly associated with degrees of tumor histological differentiation (23). Another investigation into the expression of LAMP1 in breast cancer revealed the correlation between LAMP1 expression levels and the histological grade of tumors (18). The correlation between LAMP1 expression and cell differentiation was previously reported. Another study pointed to the upregulation of LAMP1 and several lysosomal enzymes during the differentiation of keratinocytes. Based on the results of the mentioned study, the concomitant increase of LAMP1, as well as galectin 3-and -7, with keratinocyte confluence may reflect a possible role in epidermal differentiation (24, 25). Therefore, it is supposed that lysosome and their ingredient are involved in epidermal differentiation.

Furthermore, the finding of the current study signified the association between LAMP1 expression and the metastatic potential of OSCC. The cell surface expression of LAMP1 has been demonstrated to be correlated with the metastatic function of human colon carcinoma, ESCC, and breast carcinoma (12,18,23). According to the results of a previous study, the decreased metastasis of melanoma cells to the lung was observed by the downregulation of LAMP1 expression (26,27). It has been pointed out that purified LAMP1 binds to extracellular matrix (ECM) and basement membrane (BM) components, such as fibronectin, laminin, collagen-I, and IV (28). On the cytoplasmic end, LAMP1 demonstrated an interaction with ezrin which acts as a linker between the actin cortical cytoskeleton and different proteins. This might influence cellular properties that are important for motility (29-31). It has been also proposed that LAMP1 facilitates the metastasis of cancers by acting as a ligand for galectin 3 and can increase translocation to the cell membrane (32). 

To the best of our knowledge, it is the first report revealing the expression of LAMP1, which plays a prognostic role in OSCC. Among the notable limitation of the present study, we can refer to the use of normal epithelium adjacent to the tumoral tissue as a control group. In this regard, further studies are recommended to gain a better understanding of the molecular mechanism of LAMP1 in the pathogenesis of OSCC. 

## Conclusion

As evidenced by the results of the present study, the expression of LAMP1 was associated with tumor histological differentiation and metastasis. Therefore, LAMP1 might be used as a novel marker in patients with OSCC. 
